# Dissecting gastric cancer heterogeneity and exploring therapeutic strategies using bulk and single-cell transcriptomic analysis and experimental validation of tumor microenvironment and metabolic interplay

**DOI:** 10.3389/fphar.2024.1355269

**Published:** 2024-06-19

**Authors:** XianTao Lin, Ping Yang, MingKun Wang, Xiuting Huang, Baiyao Wang, Chengcong Chen, Anan Xu, Jiazuo Cai, Muhammad Khan, Sha Liu, Jie Lin

**Affiliations:** ^1^ Department of Radiotherapy, The First Affiliated Hospital of Hainan Medical University, Haikou, China; ^2^ Department of Radiation Oncology, Affiliated Cancer Hospital and Institute of Guangzhou Medical University, Guangzhou, China

**Keywords:** gastric cancer, tumor microenvironment, cancer metabolism, M2 macrophage, single-cell analysis

## Abstract

Gastric cancer, the fifth most prevalent cancer worldwide, is often diagnosed in advanced stages with limited treatment options. Examining the tumor microenvironment (TME) and its metabolic reprogramming can provide insights for better diagnosis and treatment. This study investigates the link between TME factors and metabolic activity in gastric cancer using bulk and single-cell RNA-sequencing data. We identified two molecular subtypes in gastric cancer by analyzing the distinct expression patterns of 81 prognostic genes related to the TME and metabolism, which exhibited significant protein-level interactions. The high-risk subtype had increased stromal content, fibroblast and M2 macrophage infiltration, elevated glycosaminoglycans/glycosphingolipids biosynthesis, and fat metabolism, along with advanced clinicopathological features. It also exhibited low mutation rates and microsatellite instability, associating it with the mesenchymal phenotype. In contrast, the low-risk group showed higher tumor content and upregulated protein and sugar metabolism. We identified a 15-gene prognostic signature representing these characteristics, including CPVL, KYNU, CD36, and GPX3, strongly correlated with M2 macrophages, validated through single-cell analysis and an internal cohort. Despite resistance to immunotherapy, the high-risk group showed sensitivity to molecular targeted agents directed at IGF-1R (BMS-754807) and the PI3K-mTOR pathways (AZD8186, AZD8055). We experimentally validated these promising drugs for their inhibitory effects on MKN45 and MKN28 gastric cells. This study unveils the intricate interplay between TME and metabolic pathways in gastric cancer, offering potential for enhanced diagnosis, patient stratification, and personalized treatment. Understanding molecular features in each subtype enriches our comprehension of gastric cancer heterogeneity and potential therapeutic targets.

## Introduction

Based on the 2022 estimates provided by GLOBOCAN, gastric cancer is ranked as the fifth most common malignant cancer (968, 784 new cases) and the fifth leading cause of cancer-related deaths (660, 175 deaths) worldwide (Ferlay et al., 2024). The highest numbers of cases were found in Asia, particularly in China, where approximately 820,000 new cases and 580,000 deaths were reported (Sung et al., 2021). The most common histological type of gastric cancer is stomach adenocarcinoma (STAD), accounting for 95% of cases (Sung et al., 2021; Thrift et al., 2023; Ferlay et al., 2024). Unfortunately, STAD is often diagnosed at an advanced stage in 65% of cases due to the lack of or impracticality of early detection screening strategies (Thrift et al., 2023; Ajani et al., 2017). For early-stage STAD, surgical resection and adjuvant therapy are offered, but there is a 40% chance of relapse within the first 2 years after surgery (Saito et al., 2006). Managing advanced STAD requires a multidisciplinary approach, involving surgery, chemotherapy, radiotherapy, and targeted molecular agents (Badgwell, 2016; Ajani et al., 2017). The overall 5-year survival rate for STAD patients is only 31%, increasing to 67% when diagnosed before metastasis (Howlader et al., 2012). Recent technological advancements have revealed novel mechanistic strategies that have revolutionized the therapeutic landscape for gastric cancer (Shen and Wang, 2022; Takei et al., 2022). However, the benefits of these approaches, namely, molecular targeted therapy and immunotherapy, are confined to a small subset of patients.

The advancement and widespread adoption of genomic sequencing technology have led to a significant shift in the classification of gastric cancer, moving from histological categorization to a molecular-based approach (The Cancer Genome Atlas Research Network, 2014; Cristescu et al., 2015; Oh et al., 2018). This molecular classification now holds great importance for diagnosis, prognosis, and predicting therapeutic response. However, despite this improved understanding of the molecular subtypes, the characterization of the tumor microenvironment (TME) in gastric cancer remains poorly defined. The TME is a complex and heterogeneous entity that includes both tumor and non-tumor components, such as the extracellular matrix, the network of blood vessels, secreted signaling molecules, and various infiltrated immune and stromal cells (Ren et al., 2021). The composition of the TME has been recognized as critical for tumor progression and plays a crucial role in determining the response to therapy (Fridman et al., 2017). In particular, the infiltration of diverse immune and stromal cells has shown associations with gastric cancer prognosis and clinical outcomes (Jiang et al., 2018a; Chen et al., 2013; Grunberg et al., 2021). Moreover, the characterization of TME in the context of immune and stromal cells proportions have also led to identification of TME-based GC subtypes with prognostic and therapeutic implications (Cho et al., 2018; Chen et al., 2022; Han et al., 2022). Nevertheless, these subtypes predominantly consist of a mixture of tumor and non-tumor components in varying proportions. Therefore, conducting a comprehensive exploration specifically focused on the features of the gastric cancer TME has the potential to open up critical and promising research avenues for more effective diagnosis and treatment of this disease.

Metabolic reprogramming, a cancer hallmark, fuels tumor development via enhanced proliferation and apoptosis evasion (Pavlova and Thompson, 2016). The “Warburg effect,” upregulated aerobic glycolysis in cancer cells, meets high energy needs for rapid division regardless of oxygen availability (Li and Zhang, 2016). Elevated levels of fumaric acid and alpha-ketoglutaric acid, intermediate products of aerobic glycolytic pathway, were identified in gastric tissues using gas chromatography/mass spectrometry (GC/MS) indicating the role of Warburg effect in gastric cancer (Song et al., 2011). Altered amino acid metabolism satisfies nutritional demands, links to an immunosuppressive tumor microenvironment, and drives drug resistance (Tabe et al., 2019). Proline metabolism influences cancer cell plasticity, heterogeneity, and development (D Aniello et al., 2020). The co-expression of glutaminase 1 (GLS1) and gamma-glutamylcyclotransferase (GGCT), constituents of glutamine metabolism, was strongly associated with histological grade, lymph node metastasis, and TNM stage Ⅲ/Ⅳ of gastric cancer (Jiang et al., 2019a). Reprogrammed metabolism of fatty acids, ketone bodies, and choline has significant implications for cancer diagnosis and treatment (Glunde et al., 2006; Koundouros and Poulogiannis, 2020). Fatty acid metabolic reprogramming mediated by phosphatidylinositol transfer protein, cytoplasmic 1 (PITPNC1) in adipocytes was shown to promote gastric cancer omental metastasis (Tan et al., 2018). Numerous studies have identified metabolic heterogeneity in gastric cancer through transcriptomic profiling (Zhu et al., 2021; Chen et al., 2023; Tao et al., 2023). This heterogeneity is manifested in various ways, including genetic mutations, immune cell infiltration, and prognostic implications. While the majority of research on metabolic reprogramming has focused on cancer cells, this study aims to investigate the intricate interactions between factors in the TME and metabolic activities within the context of gastric cancer’s TME.

## Materials and methods

### Transcriptomic data and processing

The transcriptional and clinical information for stomach adenocarcinoma tissues was sourced from the TCGA Data portal (https://portal.gdc.cancer.gov/repository/) for the TCGA STAD experimental cohort (n = 407), while for internal validation (GEO ID: GSE84437; n = 433) and external validation (GEO ID: GSE15459; n = 192), data was retrieved from the GEO database (https://www.ncbi.nlm.nih.gov/geo/). The baseline characteristics of participants for these cohorts are detailed in Supplementary Table S1. To preprocess the datasets, log normalization was performed using the ‘limma’ R package. Furthermore, batch effects were removed using the ‘sva’ R package and the “Combat” function.

### Single cell data quality control and processing

We acquired the GSE112302 single-cell sequencing data from the GEO database, and then used the “Seurat” R package for quality control (Slovin et al., 2021). Cells were filtered based on feature RNA counts (>50) and the percentage of mitochondrial gene expression (<5%). The “NormalizeData” function was applied with the “LogNormalize” method and a scale factor of 10,000. We employed the “FindVariableFeatures” function to identify the top 2000 variable features using the “vst” method. These features were then used for principal component analysis through the “ScaleData” and “RunPCA” functions. Subsequently, we performed cell clustering using the k-nearest neighbor classification (KNN) algorithm via the “FindNeighbors” and “FindClusters” functions, with a resolution of 1.0. For dimensionality reduction, we used the “RunUMAP” function and visualized the results using Uniform manifold approximation and projection (UMAP) plots. Cell markers were identified by utilizing the “FindAllMarkers” function. The normalized count matrix of another GC single cell dataset (GSE167297) was downloaded from TISCH website (Sun et al., 2021). Count matrix was preprocessed for quality control according to the standard pipeline in MAESTRO (Wang et al., 2020). A similar processing steps as mentioned above were taken for generating principal components (PCA) and performing dimensionality reduction with 40 dimensions and resolution of 0.5.

### Identification of TME- and metabolism-related genes

We acquired a comprehensive collection of 4,061 genes associated with the tumor microenvironment (TME) from a selection of prior research studies (Newman et al., 2015; Rooney et al., 2015; Becht et al., 2016; Chifman et al., 2016; Aran et al., 2017; Tirosh et al., 2016). These TME-related genes encompass a fusion of TME genes consolidated from three prominent TME algorithm databases (xCell, CIBERSORT, and MCP-counter) (Newman et al., 2015; Aran et al., 2017; Becht et al., 2016), immune gene signatures derived from two substantial studies encompassing multiple cancer datasets (Rooney et al., 2015; Chifman et al., 2016), and a single-cell melanoma dataset with a specific focus on TME elements (Tirosh et al., 2016). Metabolism-related genes (n = 945) were acquired from the Molecular Signatures Database (https://www.gsea-msigdb.org/gsea/msigdb/index.jsp) (Supplementary Table S2). These metabolism-related genes are derived from gene signatures related to KEGG (Kyoto Encyclopedia of Genes and Genomes) pathways associated with metabolic processes.

### Delineation of TME- and metabolism-related genes crosstalk

Differential expression of TME- and metabolism-related genes (TME-Met DEGs) between the TCGA STAD normal (n = 32) and tumor samples (n = 375) were characterized by the following criteria: log foldchange (logFC) = 1, and false discovery rate (FDR) < 0.05. Next, univariate cox regression analysis was carried out to estimate the prognostic significance of the TME-Met DEGs (TME = 1,021; Met = 147 DEGs). Significance level was set at *p* < 0.05. Search Tool for the Retrieval of Interacting Genes (STRING) database, version 11.5 (https://string-db.org/) was used to evaluate protein-protein interactions (PPI) among the prognostically significant TME-Met DEGs (TME = 121; Met = 20 DEGs). Interactions score was set at 0.4.

### Non-negative Matrix Factorization (NMF) clustering

The expression data of 81 TME-Met prognostic DEGs with significant interaction at protein level were subjected to clustering using the Non-negative Matrix Factorization (NMF) algorithm. The ‘brunet’ criterion was employed, and 100 iterations were performed. The range of cluster numbers (k) was set from 2 to 10. The average contour width of the common membership matrix was calculated using the ‘NMF’ package in R (Gaujoux and Seoighe, 2010). The cluster stability resulting from NMF was assessed using the cophenetic correlation, which ranged from 0 to 1. A higher value indicated greater cluster stability. Additionally, smaller values of the residual sum of squares (RSS), used to gauge the clustering performance of the model, suggested better clustering performance. The optimal number of clusters was determined based on metrics such as cophenetic correlation, dispersion, and silhouette. By employing the above algorithm, the samples were categorized into distinct molecular subtypes.

### Development and validation of TME-Met prognostic model

To construct the prognostic model for the upregulated TME-Met DEGs (n = 47) within molecular subtype 2 (C2), we employed the “glmnet” R package. We conducted a Least Absolute Shrinkage and Selection Operator (LASSO) penalized Cox regression analysis to systematically identify the most relevant candidate genes (Ishwaran et al., 2014). Our regression analysis utilized the normalized expression matrix of the candidate prognostic DEGs as independent variables. Simultaneously, the overall survival data and patient survival status from the TCGA STAD cohort were employed as response variables. To optimize the model, we determined the penalty parameter (λ) through a tenfold cross-validation approach. We selected the λ value that minimized the partial likelihood deviance. Subsequently, we calculated a risk score for each patient based on the expression levels of the DEGs and their corresponding coefficients. This risk score was computed using the following formula:Risk Score=(Expression of mRNA1×Coefficient mRNA1)+(Expression of mRNA2×Coefficient mRNA2)+…+(Expression of mRNAn×Coefficient mRNAn).Risk Score=(Expression of mRNA1×Coefficient mRNA1)+(Expression of mRNA2×Coefficient mRNA2)+…+(Expression of mRNAn×Coefficient mRNAn). The median risk score was used to classify patients into low- and high-risk cohorts. Kaplan-Meier analysis was performed to compare overall survival between the risk groups. The optimal cut-off expression value was determined using the “surv_cutpoint” function from the “survminer” R package. To assess the predictive power of the gene signature, time-dependent ROC curve analyses were conducted using the “survivalROC” R package. These steps were repeated for internal and external validation in the GEO cohorts.

### Independent prognostic analysis

In order to examine the independent prognostic factors of gastric adenocarcinoma, including variables such as age, gender, tumor grade, and tumor stage (TNM staging data), univariate Cox regression and multivariate Cox regression analyses were performed on genes using the R software forestplot package. The results were displayed using forest plots to visualize the findings. A factor was considered an independent prognostic factor when its *p*-value was less than 0.05 in both the univariate Cox and multivariate Cox analyses.

### Immunological analysis

The MCP-counter algorithm was utilized to assess the primary cellular constituents of the TME (Becht et al., 2016). This algorithm robustly calculates the absolute abundance of immune cells (eight distinct types) and stromal cells (two types) by analyzing the transcriptomic data from heterogeneous tissues. The CIBERSORT algorithm was employed for a quantitative analysis of the relative abundance of 22 immune cell types, encompassing various subtypes of major cell lineages, within the TCGA cohort (Newman et al., 2015). CIBERSORT is a computational method designed to estimate cellular fractions by utilizing gene expression profiles from bulk cancer tissues. The “ESTIMATE” R package was utilized to infer the tumor purity and immune and stromal cell admixture from cancer tissue gene expression data (Yoshihara et al., 2013). Additionally, to determine the immune subtype of the risk groups, we referred to previous research for immune subtype information and analyzed the enrichment of each subtype in the high- and low-risk groups (Thorsson et al., 2018).

### Functional enrichment analysis

The “clusterProfiler” package was utilized to examine the enrichment of KEGG pathways by 81 TME-Met DEGs and DEGs between risk groups, as well as the enrichment of Gene Ontology (GO) terms by 15 risk signature genes. The “gsva” package of R was used to estimate the enrichment of the hallmark cancer and KEGG pathways operating between the clusters.

### Evaluation of genetic alterations

We acquired the Simple Nucleotide Variation data of the TCGA STAD cohort from the University of California, Santa Cruz (UCSC) Xena website (https://xenabrowser.net/). For the analysis of gene mutations and the creation of oncoplots, we employed the R package “maftools”. To calculate somatic copy number alterations (SCNAs) at both the arm and focal levels in the tumor, we utilized GISTIC_2.0. The input for this analysis consisted of “SNP6" files, which were downloaded from the genomic data commons data portal (https://portal.gdc.cancer.gov/) (Mermel et al., 2011).

### Cell lines and cell culture

The human gastric cancer cell lines (AGS and MKN45) and normal gastric cell line of GSE-1 were acquired from the Committee of Type Culture Collection of the Chinese Academy of Sciences (Shanghai, China). The cells were cultured in DMEM medium supplemented with 10% fetal bovine serum (FBS), penicillin (100 U/mL), and streptomycin (100 mg/mL). A humid incubator set at 37°C and 5% CO2 was used to maintain the cells at 37°C. We regularly performed authentication checks on all cell lines utilized in this investigation by assessing their morphology and conducted tests to ensure the absence of *Mycoplasma* contamination.

### Quantitative real-time PCR

The extraction and purification of total RNA were carried out using Trizol Reagent (Takara, Otsu, Japan), followed by reverse transcription to generate cDNA. Quantitative real-time polymerase chain reaction (qRT-PCR) was performed using a SYBR Green PCR Kit (Takara, Otsu, Japan). The expression of mRNA was normalized to the internal control Beta Actin, and the relative mRNA level of the treated group was compared to the control group. The primer sequences utilized in this study were generated using the primer-BLAST tool (https://www.ncbi.nlm.nih.gov/tools/primer-blast/index.cgi?GROUP_TARGET=on) and obtained from Tsingke Biotechnology Co., Ltd. The primer sequences can be found in Supplementary Table S3.

### Immunohistochemistry analysis

Deparaffinization of formalin-fixed, paraffin-embedded 4-mm thick tumor tissue sections was carried out using xylene and ethanol. Subsequently, antigen retrieval was performed by boiling the sections in a microwave oven using citrate buffer (pH 6.0). This was followed by blocking of endogenous HRP activity with 0.3% hydrogen peroxide. Following the washing step with 10% phosphate-buffered saline (PBS), the sections were blocked using 5% BSA and then exposed to primary antibodies against the following targets: KYNU (Proteintech, #11796-1-AP, Rabbit, 1:100), CPVL (Proteintech, #12548-1-AP, Rabbit, 1:200), CD36 (Proteintech, #18836-1-AP, Rabbit, 1:800), GPX3 (Signalway Antibody, #27854, Rabbit, 1:800), and CD163 (Cell Signaling, #93498S, Rabbit, 1:250). This incubation process took place at 4°C overnight. Afterward, the sections were subjected to a 20-min incubation with a biotinylated goat anti-rabbit IgG secondary antibody at room temperature. The visualization of the targeted proteins was achieved using the 3,5-diaminobenzidine (DAB) Substrate Kit, and finally, counterstaining with Hematoxylin was performed. The staining intensity was assessed using a semi-quantitative method with the following scores: 0 for negative, 1 for weak, 2 for moderate, and 3 for strong. Additionally, the frequency of positive cells was categorized as follows: 0 for less than 5%, 1 for 5%–25%, 2 for 26%–50%, 3 for 51%–75%, and 4 for greater than 75%. The final Immunohistochemistry (IHC) scores were obtained by multiplying the staining intensity and the frequency of positive cells. In cases of heterogeneous tissue staining, each area was scored independently, and the scores were then combined to determine the final result. The study adhered to ethical guidelines, with patient informed consent obtained, and approval from the internal review and ethics boards of the Affiliated Cancer Hospital and Institute of Guangzhou Medical University was obtained as well (Approval Number: GYZL-ZN-2023(029)).

### Therapeutic response assessmsent

The Tumor Immune Dysfunction and Exclusion algorithm (TIDE) (http://tide.dfci.harvard.edu) was utilized to assess the immunotherapy response (Jiang et al., 2018b). An analysis of drug sensitivity was conducted on 198 small molecules. To calculate the half-maximal inhibitory concentration (IC50) of these drugs in GC patients, the “oncoPredict” R package was employed (Maeser et al., 2021). An effective drug for the patient is indicated when the IC50 value of the high-risk group is lower than that of the low-risk group, and there is a significant difference between the two risk groups. To further identify sensitive drugs with a low and differentiated IC50 in the high-risk group, this information was combined with the results obtained from the risk model.

### Experimental assessment of drug inhibitory effect in gastric cancer

We conducted a CCK-8 assay using the MKN45 and MKN28 cell lines. The cells were first washed three times with phosphate-buffered saline (PBS) and then treated with 2 mL of trypsin for 1–2 min. Subsequently, the trypsin was aspirated, and the cells were gently detached using a disposable pipette. The dislodged cells were transferred to a centrifuge tube containing 2 mL of culture medium to create a single-cell suspension. Cell counting was performed by placing 10 μL of the single-cell suspension on a hemocytometer plate, and the concentration of the suspension was adjusted to 5 × 104 cells/mL. After 24 h, we removed the 96-well plates and added 100 μL of a fresh drug solution with various concentrations (0, 10, 50, 100, 200, 500 μmol/L) to each well. Each group included three replicate wells, and the plates were incubated in a standard cell culture incubator. The assessment of the drug’s inhibitory effect on MKN45 and MKN28 cells was determined by measuring the optical density (OD) values on days 1 and 2 after the addition of the drug solution.

### Statistical analysis

The gene expression level and drug sensitivity between the groups were assessed using the Wilcoxon test. Categorical variables were compared using the Chi-square test. To estimate the difference in overall survival between the groups, the Kaplan-Meier method with the log-rank test was utilized. Univariate and multivariate factor analyses were conducted using cox-regression hazard models. All statistical analyses were executed using R software (v4.0.2).

## Results

### Data processing and the analysis pipeline

**FIGURE 1:**
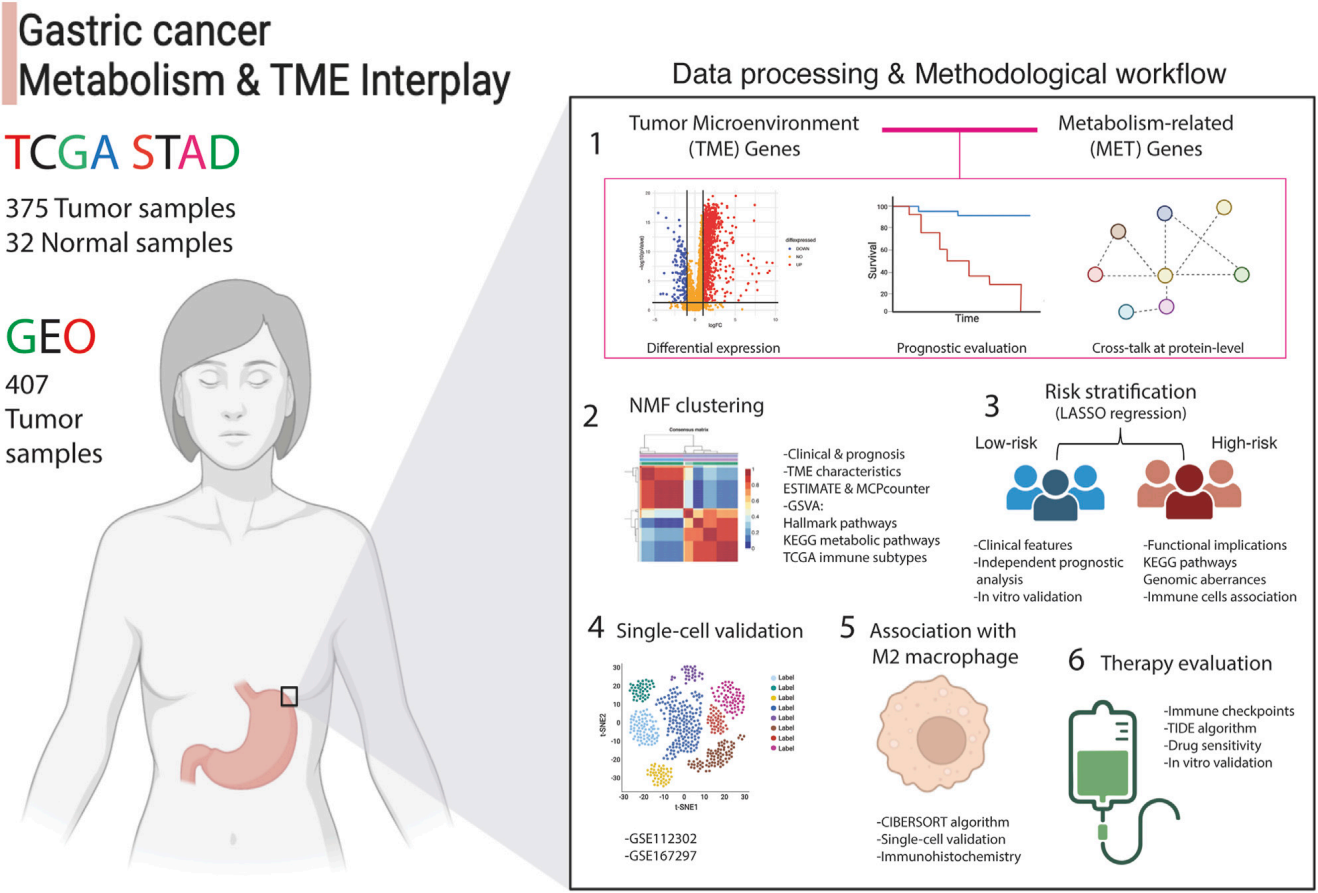
Analytical process adopted in this article. The interplay between tumor microenvironment and metabolism genes was examined using transcriptomic data from 375 stomach adenocarcinoma (STAD) patients from TCGA (training set) and 407 tumor samples (validation set) from the GEO database. Differential expression analysis compared tumor (n = 375) to normal samples (n = 32), followed by uni-cox regression analysis and protein-level interaction analysis. This identified 81 crosstalk genes, labeled as TME-Met genes, which were then subjected to NMF clustering to assess clinical and functional implications. LASSO regression yielded a 15-gene risk signature, termed TME-Met risk signature, to stratify STAD patients into high and low-risk categories. Clinical, functional, and immunological features were evaluated, and gene expression in gastric cancer cells was validated using RT-qPCR. Single-cell validation identified four genes predominantly expressed by M2 macrophages, confirmed via IHC analysis. Finally, therapeutic implications were explored, identifying three potential drugs for inhibiting gastric cell proliferation.

A depiction of the data processing and the systematic analytical approach employed in this article is presented in Figure 1. Transcriptomic data of stomach adenocarcinoma samples from TCGA and GEO databases served as the input datasets. A thorough six-step analysis was conducted to investigate the interplay between components of the Tumor Microenvironment (TME) and metabolic influences within the TME of gastric cancer (Figure 1).

### Discovering prognostic DEGs associated with tumor microenvironment and metabolism, and their protein-level interactions

**FIGURE 2:**
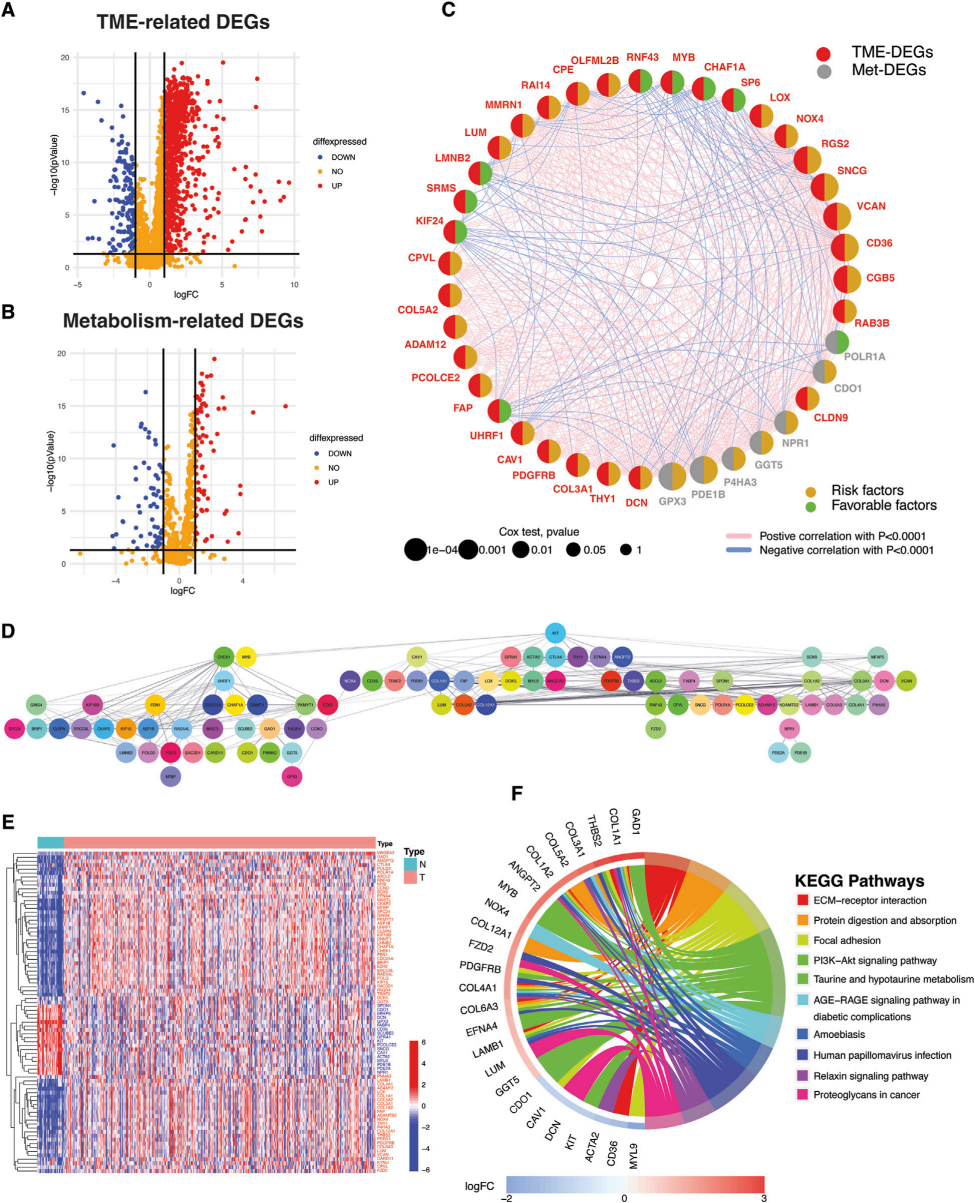
Identification of TME- and metabolism-related DEGs with protein-level interactions and prognostic significance. **(A)** Volcano plots depicting TME- and **(B)** metabolism-related differentially expressed genes (DEGs). DEGs were defined according to the following criteria: log fold change (logFC) = 1, and the false discover rate (FDR) < 0.05. **(C)** Bubble Network illustrating the prognostic impact of significant TME- and metabolism-related DEGs and correlation among them. **(D)** Protein-protein interaction (PPI) network depicting protein-level interactions of TME-Met DEGs at interaction score = 0.4. **(E)** Heatmap shows the expression pattern of TME-Met DEGs between TCGA STAD normal (n = 32) and tumor samples (n = 375). Red and blue represent upregulation and downregulation respectively. **(F)** Circos plot depicting KEGG pathway enrichment analyses of TME-Met DEGs. (increasing depth of the red indicate the more obvious differences; q-value: the adjusted *p*-value).

Comparison of TCGA STAD normal (n = 32) and tumor tissues (n = 375) revealed differential expression of 1021 TME- and 147 metabolism-related genes as illustrated in the volcano plots (Figures 2A,B; Supplementary Table S4, S5). Out of the 1021 TME DEGs, 121 genes (48 protective and 73 risky genes) were found to be significantly associated with TCGA STAD prognosis (Supplementary Table S6). Similarly, 13 risky and 7 protective metabolism-related genes were also identified, showing significant associations with prognosis (Supplementary Table S7). A TME-Met (Tumor Microenvironment-Metabolic) prognostic network was constructed to illustrate the correlations among these genes and their prognostic significance (Figure 2C). Prognostic genes were further validated for protein-level interactions using the STRING database (Figure 2D). A subset of 81 genes showed significant protein-level interactions. These genes, drawn from both the TME and metabolism groups, exhibit confirmed cross-talk at the protein level and are linked to gastric cancer prognosis. They were selected for further analysis as they constitute the desired gene set for in-depth exploration into the interplay between TME and metabolism in gastric cancer. The heatmap visualizes the differential expression of these genes in the TCGA STAD cohort, with 18 of them being downregulated in cancer tissues compared to adjacent normal tissues (Figure 2E). The enrichment analysis of KEGG pathways revealed that the identified genes are actively involved in essential pathways that are fundamental to the tumor microenvironment’s functioning and the associated metabolic processes (Figure 1F; Supplementary Table S8). This finding suggests that these genes may play critical roles in shaping the TME and influencing the metabolic alterations that occur during cancer development and progression.

### Clustering analysis reveals distinct molecular subgroups driven by TME-Metabolic interactions

**FIGURE 3:**
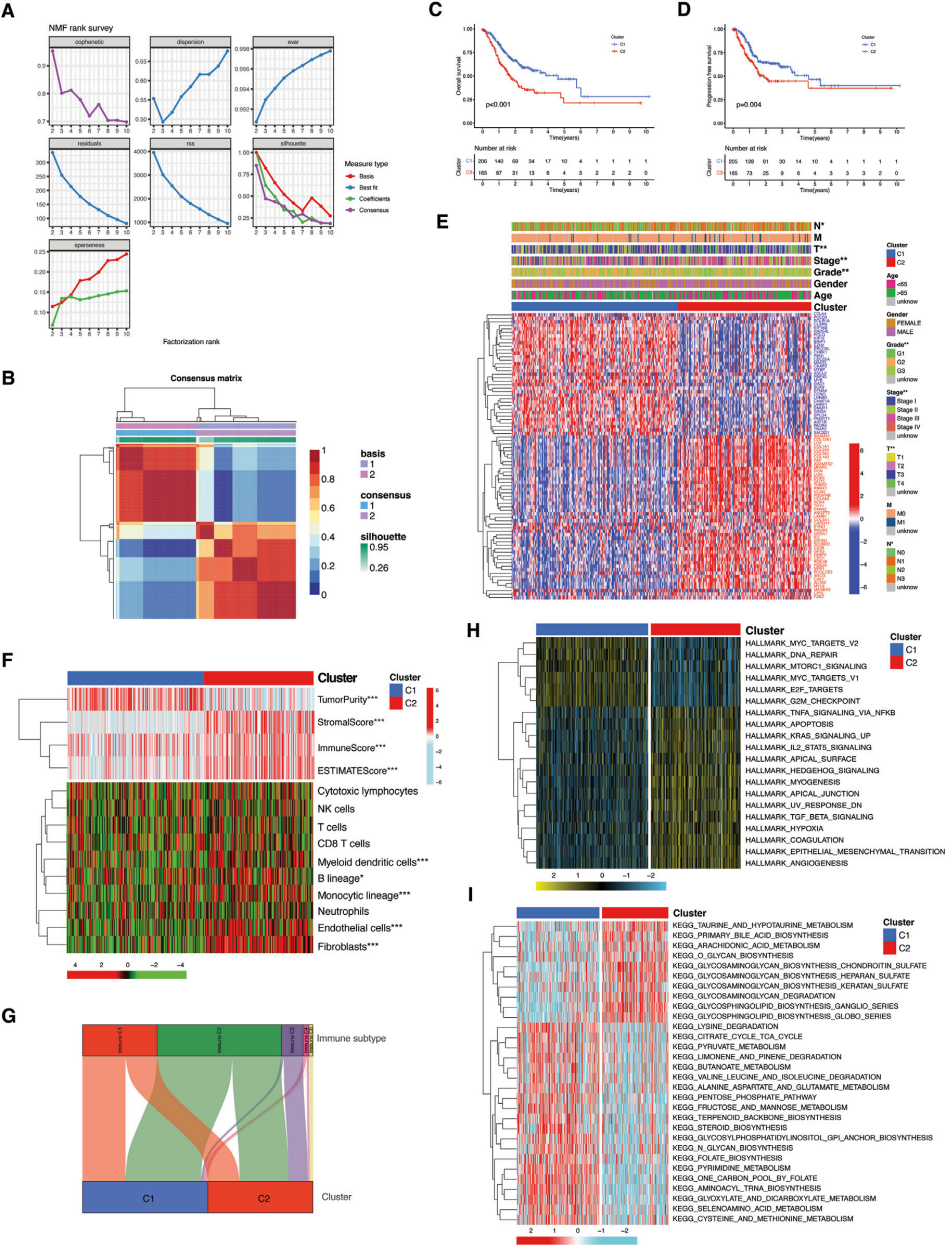
Molecular subtyping and functional implication of TME-Met DEGs cross-talk. **(A, B)** Consensus clustering matrix in TCGA STAD patients. **(C)** Kaplan-Meier curves for the OS and **(D)** PFS comparison between clusters. **(E)** Heatmap illustrating the expression of 81 TME-Met DEGs (Red: upregulation; Blue: downregulation) between the clusters and correlation between clusters and clinicopathological features. **(F)** Heatmap shows the enrichment results of ESTIMATE algorithm and single-sample gene set enrichment analysis (ssGSEA) of immune cell infiltration between the clusters. *p* values are shown as: *p < 0.05; **p < 0.01; ***p < 0.001. **(H)** Sankey diagram presenting the correlation between clusters and immune subtypes. **(I)** Heatmaps showing GSVA enrichment scores of hallmark cancer pathways and **(G)** KEEG metabolic pathways in the clusters.

In order to delineate the functional and biological implications of TME-Met crosstalk genes, TCGA STAD tumor samples were stratified into molecular clusters by performing the unsupervised clustering analysis using NMF algorithm. Two molecular clusters were obtained as suggested by cophenetic correlation coefficient and visual inspection of the consensus matrix (Figures 3A,B). The C2 cluster showed significantly lower PFS and OS as shown in Kaplan-Meier curves (Figures 3C,D). Differential expression of these genes between the clusters and their association with clinical features is highlighted in Figure 3E, which reveals the association of C2 with more aggressive clinical features of STAD patients, such as grade, stage, T and N stage. According to the ESTIMATE algorithm, the TME (non-tumor components) was strongly represented in the C2 cluster, whereas the C1 cluster exhibited a higher concentration of tumor components (Figure 3F). The most noticeable dissimilarity between the clusters was the enrichment of the stromal component in C2. Immunologically, C2 was infiltrated by myeloid dendritic cells, monocytic lineage cells, endothelial cells, and fibroblasts (Figure 3F). No difference in cytotoxic and adaptive immune cells was observed between clusters. Correspondingly, there was no variation between the clusters in their correlation with immune subtype C1 (wound healing) and C2 (IFN-γ dominant) (Figure 3G). However, the C3 and C6 immune subtypes were predominantly associated with the C2 cluster, representing the primary distinction between the clusters. Functional evaluation via Hallmark pathways demonstrated that C1 was enriched in cell division, DNA repair, and oncogenic pathways corresponding to tumor cells (Figure 3H; Supplementary Table S9). While C2 was predominantly enriched in inflammatory, metabolic, immunosuppressive, and pathways associated with cancer progression and invasion. The difference in metabolic activity, as assessed by the enrichment of KEGG metabolic pathways, further confirmed these distinctions. The C2 cluster exhibited heightened metabolism of glycosaminoglycans (GAGs) and glycosphingolipids (GSLs), while pathways linked to protein and sugar metabolism were predominantly enriched in C1 (Figure 2I; Supplementary Table S10). In brief, these findings imply that C2 is predominantly composed of elements linked to the tumor microenvironment (TME), while C1 shows enrichment in characteristics associated with tumors. Additionally, the two clusters display substantial differences in metabolic activity, carrying clinical and prognostic significance.

### Establishing and validating the TME-Met Interplay prognostic index

**FIGURE 4:**
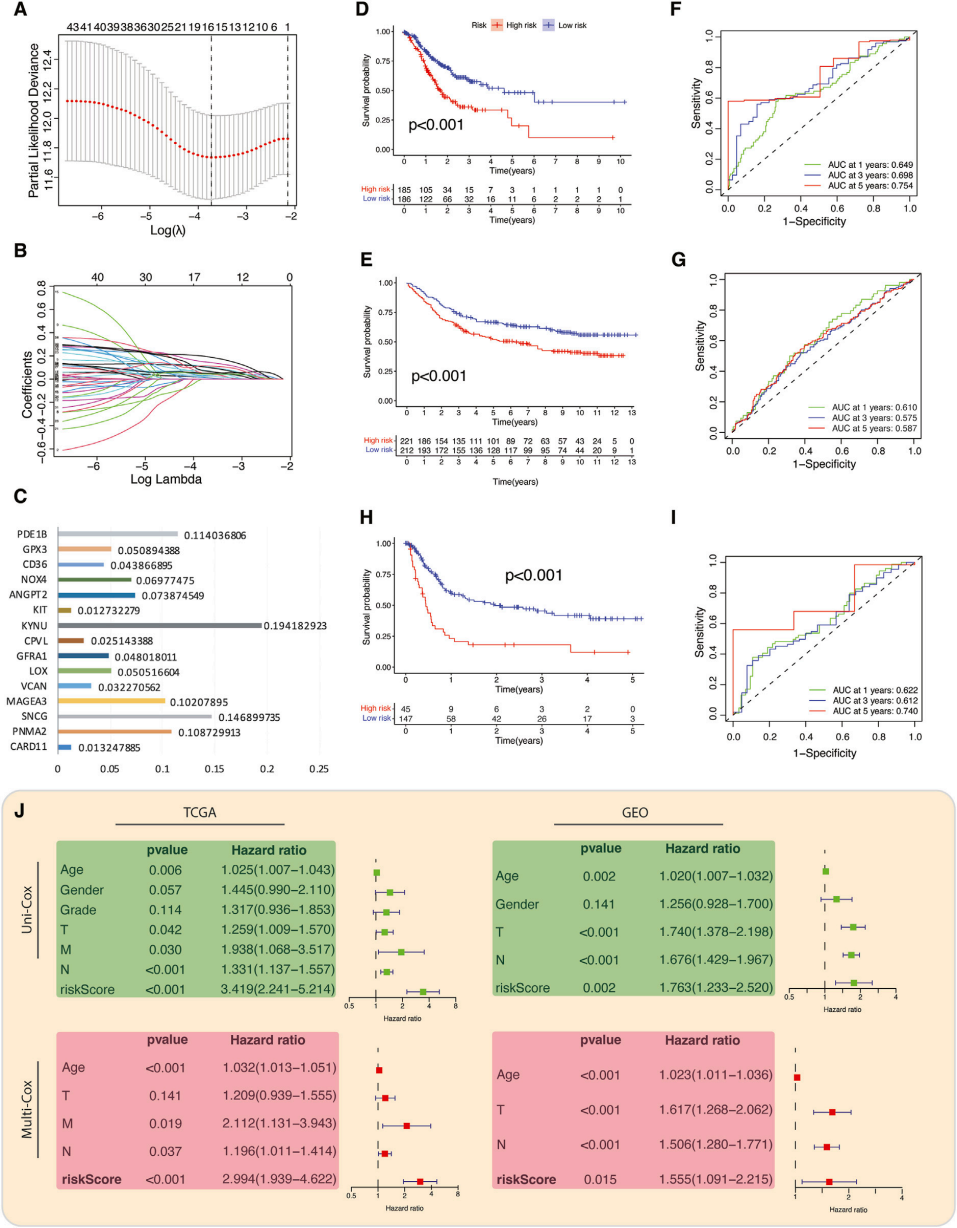
Construction and validation of TME-Met prognostic index. **(A)** LASSO regression of the 81 TME-Met genes and **(B)** Cross-validation for tuning the parameter selection in the LASSO regression. **(C)** Bar plot depicting the regression coefficients of the 15 TME-Met prognostic index (PI) genes. **(D)** Kaplan-Meier curves for the OS difference between risk subgroups in the TCGA cohort and **(E)** GEO cohort. **(F)** Time-dependent receiver operating characteristic (ROC) curves and area under curve (AUC) analyses depicting the predictive efficiency of riskScore in TCGA cohort and **(G)** GEO cohort. **(H)** Kaplan-Meier curves for the OS difference between risk subgroups in the TCGA cohort and **(I)** Time-dependent ROC curves and AUC analyses depicting the predictive efficiency of riskScore in external validation cohort (GSE15459). **(J)** Univariate and multivariate cox-regression analysis to evaluate the independent prognostic value of the risk score in TCGA and GEO cohorts.

We generated a prognostic model by exclusively considering the upregulated genes (referred to as risky genes) in C2 for conducting the LASSO Cox regression analysis (Figures 4A,B). A 15-gene prognostic signature was constructed, consisting of TME- and metabolism-related genes. A TME-Met prognostic index (PI) was established based on the regression coefficients of the genes: TME-Met PI = (0.114036806 * PDE1B) + (0.050894388 * GPX3) + (0.043866895 * CD36) + (0.06977475 * NOX4) + (0.073874549 * ANGPT2) + (0.012732279 * KIT) - (0.194182923 * KYNU) + (0.0251143388 * CPVL) + (0.048018011 * GFRA1) + (0.050516604 * LOX) + (0.032270562 * VCAN) + (0.10207895 * MAGEA3) + (0.146899735 * SNCG) + (0.108729913 * PNMA2) + (0.013247885 * CARD11) (Figure 4C). TCGA STAD patients were categorized into high- and low-risk subgroups based on the median TME-Met PI, and the same procedure was replicated for internal validation using a GEO dataset (GSE84437). Kaplan-Meier overall survival curves revealed a significantly worse prognosis for high-risk patients in both datasets (Figures 4D,E). The TME-Met PI demonstrated an area under the curve (AUC) of 0.649, 0.698, and 0.754 at 1-, 3-, and 5-year survival, respectively. The GEO cohort also showed promising AUC scores of 0.610, 0.575, and 0.587 at 1-, 3-, and 5-year. AUC score at 5 years was lower in GEO cohort as compared to TCGA cohort, possibly due to lack of patients with distant metastases (Figures 4F,G). Moreover, the ability of TME-Met PI to stratify GC patients’ survival probabilities was also demonstrated in an external validation cohort of 192 GC patients (GSE15459) (Figures 4H,I). Intriguingly, the AUC scores closely resembled those of TCGA STAD patients, reinforcing the notion that the TME-Met PI performs more effectively in advanced GC cases as this cohort also involved GC patients with metastatic disease (N = 60). Furthermore, we confirmed the independent prognostic value of the risk model by conducting uni- and multi-variate Cox regression analysis as shown in Figure 4J.

### Clinical and functional annotations of TME-Met prognostic index

**FIGURE 5:**
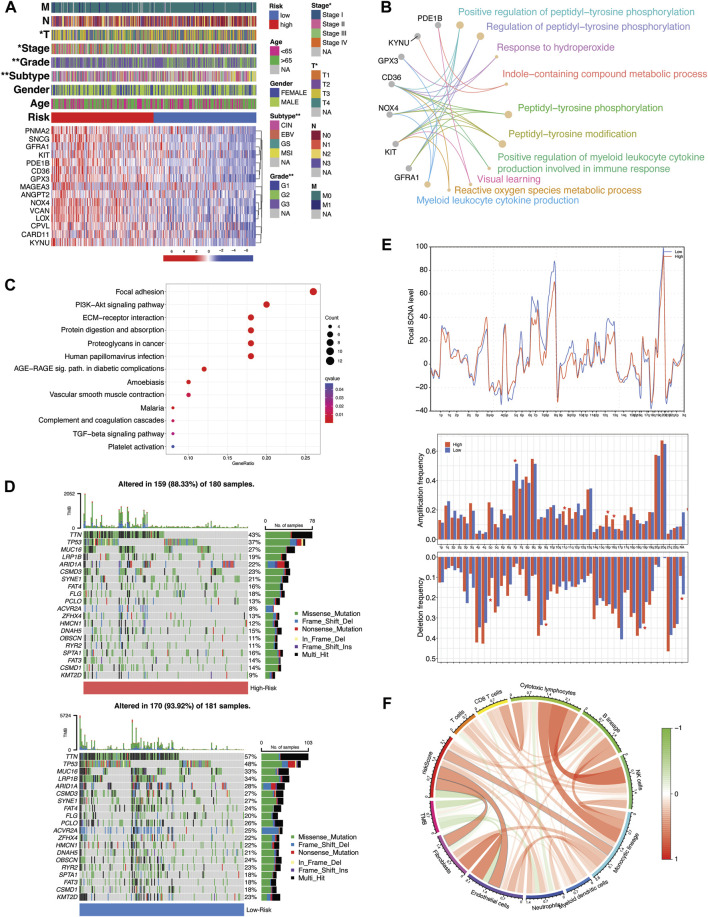
Molecular and functional implications of TME-Met prognostic index. **(A)** Heatmap illustrating the expression of 15 TME-Met PI genes (Red: upregulation; Blue: downregulation) in the TME-Met PI risk subgroups (Red: high-risk; Blue: low-risk) and association with clinicopathological features (TNM staging. T: primary tumor; N: lymph node; M: metastasis. Degree of differentiation. G1: highly differentiated; G2: moderately differentiated; G3: poorly differentiated). **(B)** KEGG pathway enrichment analyses of TME-Met PI genes and **(C)** DEGs between the risk subgroups. **(D)** Oncoplot depicting the mutation frequency of top 20 mutated genes in the high- and low-risk groups. **(E)** Comparisons of focal- and arm-level amplification and deletion frequencies levels between risk subgroups. **(F)** Correlation among riskScore, TMB and TME infiltrates.

The expression pattern of 15-risk TME-Met prognostic genes in high and low-risk subgroups and their correlation with clinical features is highlighted in Figure 5A. The TME-Met PI was able to describe the aggressive feature of STAD patients such as primary tumor size (T), tumor clinical stage and pathological grade (Figure 5A). High-risk subgroup patients had significantly high primary tumor size (T3/4 vs T1/T2), clinical stage (III/IV vs I/II), and pathological grade (G3 vs G2). Although the presence of distant metastasis was more common in high-risk subgroup, the difference was not statistically significant (17 vs 8, *p* = 0.078). Among the four GC molecular subtypes, genomic stability (GS) subtype was more common in high-risk subgroup while microsatellite instability (MSI) was abundant in the low-risk subgroup (Shen and Wang, 2022). The enrichment analysis of GO terms revealed that these genes play a significant role, either independently or in combination, in intracellular signaling, metabolic processes, and cytokine production by myeloid leukocytes (Figure 5B). Additionally, the DEGs between these risk groups also exhibited enrichment for KEGG pathways related to the tumor microenvironment (TME), including ECM-receptor interaction and Proteoglycans in cancer (Figure 5C; Supplementary Table S11). Furthermore, the pathways associated with cancer progression and immunosuppression, such as PI3K-Akt and TGF-β signaling, were also found to be enriched in these DEGs. In the low-risk subgroup, STAD-specific mutations were more common compared to the high-risk subgroup (88.33% vs 93.92%) (Figure 5D). The three most frequently mutated genes in this subgroup were TTN (43% vs 57%), TP53 (37% vs 48%), and MUC16 (27% vs 33%). Additionally, there was a noticeable increase in focal-level SCNAs in the low-risk subgroup with only few significant arm-level amplifications and deletions in the high-risk subgroup (Figure 5E). It is worth noting that the distribution of TME components (tumor *versus* non-tumor) between these risk subgroups might also play a role in the low genomic alterations seen in the high-risk subgroup, potentially influenced by a lower tumor content in that group. Moreover, our observations revealed a positive correlation between riskScore and the presence of immune and stromal cells, while there was a negative correlation with tumor mutational burden (TMB), consistent with previous research findings. (Jiang et al., 2020) (Figure 5F). Overall, the high-risk subgroup demonstrated larger tumor size, higher pathological grade, enhanced genomic stability, lower mutation rates, and an immunosuppressive TME marked by the presence of myeloid cells-derived cytokines, epithelial-to-mesenchymal transition (EMT), and an enrichment of PI3K-Akt and TGF-β signaling pathways. This profile aligns with the mesenchymal phenotype previously described in GC subtypes, characterizing the high-risk subgroup (The Cancer Genome Atlas Research Network, 2014; Cristescu et al., 2015; Oh et al., 2018).

### Profiling the dynamics of hub gene expression in gastric cancer within TME context

**FIGURE 6:**
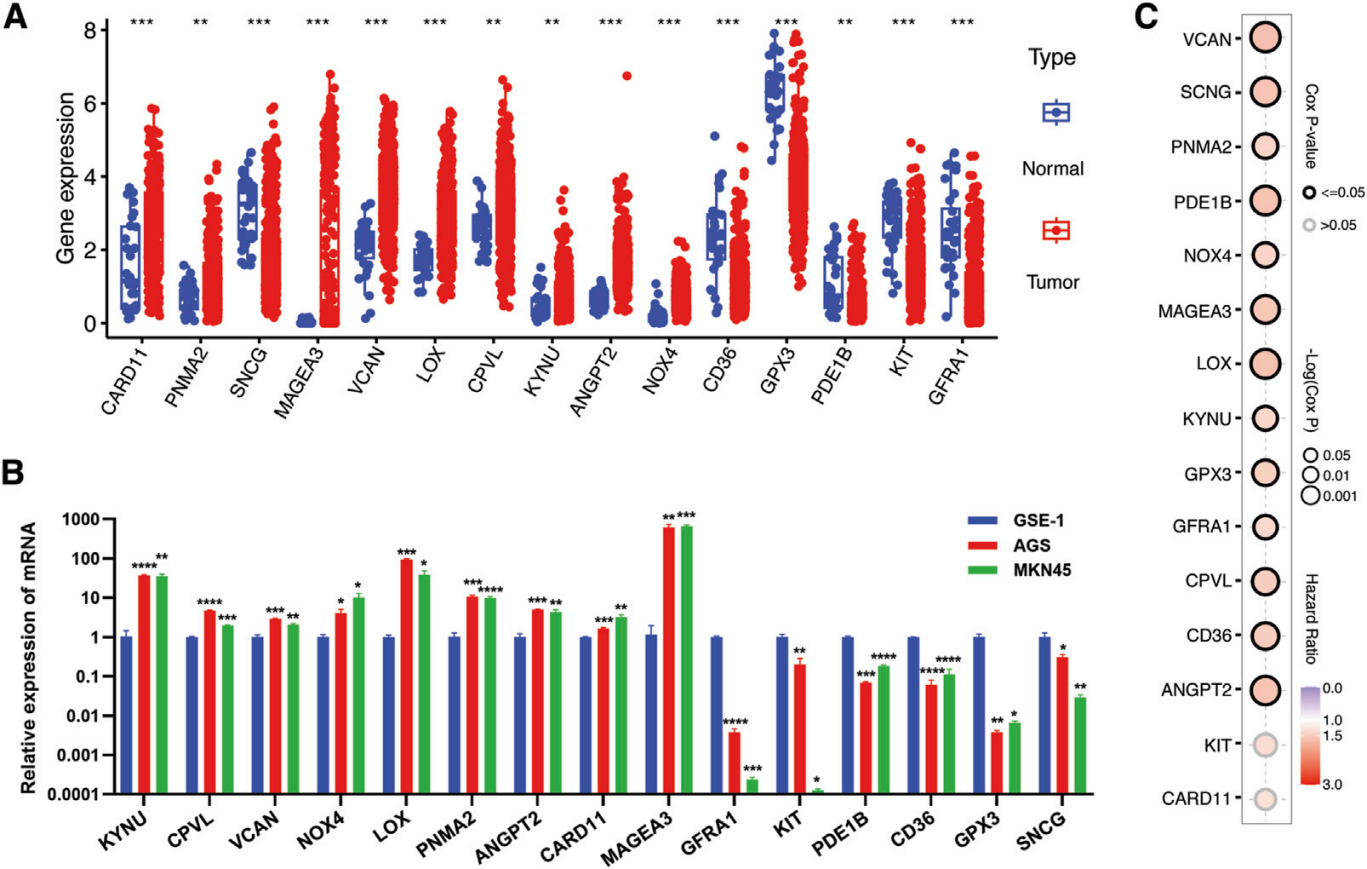
Verification of expression of TME-Met PI genes in gastric cancer. **(A)** The expression levels of TME-Met PI genes between risk subgroups in the TCGA STAD cohort. **(B)** mRNA expression level of TME-Met PI genes in gastric normal cell (GSE-1) and cancer cells (AGS and MKN45). *p* values are shown as: *p < 0.05; **p < 0.01; ***p < 0.001). **(C)** Bubble plot depicts overall survival significance of TME-Met PI genes in TCGA STAD samples.

**FIGURE 7:**
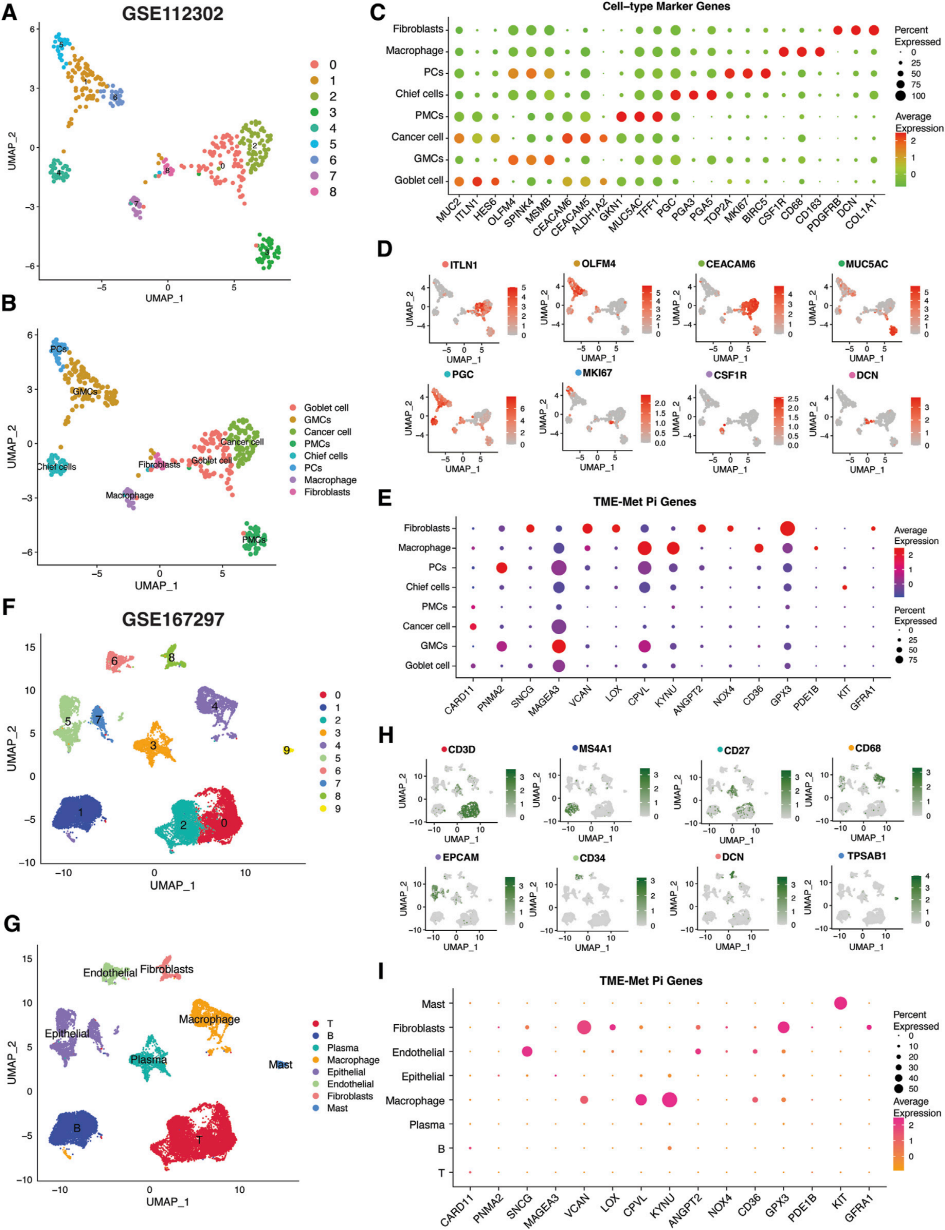
Single-cell transcriptomic analysis of TME-Met PI genes in gastric cancer. **(A, B)** Uniform manifold approximation and projection (UMAP) plots showing main clusters and cell-types in single-cell gastric cancer dataset (GSE112302), colored by cluster **(A)** and **(B)** cell type. **(C)** Bubble plot shows the expression levels of top 3 marker genes in each cell-type for GSE112302 dataset. **(D)** UMAP plots displaying expression patterns of cell-specific marker genes for each cell-type in GSE112302 dataset. **(E)** The bubble plot depicting the expression levels of TME-Met PI genes in all cell types in GSE112302 dataset. **(F, G)** UMAP plots showing main clusters and cell-types in single-cell gastric cancer dataset (GSE167297), colored by cluster **(F)** and **(G)** cell type. **(H)** UMAP plots displaying expression patterns of cell-specific marker genes for each cell-type in GSE167297 dataset. **(I)** The bubble plot depicting the expression levels of TME-Met PI genes in all cell types in GSE167297 dataset.

As mentioned in the previous sections and highlighted in Figure 6A, some of the hub genes were downregulated in the TCGA STAD cancer samples as compared to normal tissues. These genes included GFRA1, KIT, PDE1B, CD36, GPX3, and SNCG. Our *in vitro* assessment of the expression of these genes in gastric cancer (GC) cell lines (AGS, MKN45) also indicated lower expression in cancer cells compared to normal control (GSE-1) (Figure 6B). The downregulation of these genes in cancer samples does not necessarily imply that they are tumor suppressor genes, as their expression is positively correlated with poor prognosis (Figure 6C). Instead, this points to their unique expression pattern specific to a particular subset of patients, as indicated in our study. Consequently, we proceeded to investigate their expression pattern at the single-cell level using a single-sequencing dataset (GSE112302) sourced from the GEO database. This dataset encompassed 6 gastric cancer samples, comprising a total of 400 single cells and 24,000 genes. In total, 9 clusters of cell populations were obtained after the initial quality control and data standardization steps using the “Seurat” R package (Figure 7A). The clusters were categorized into 8 distinct types of cell subsets based on the expression of stomach cell markers (Figure 6B). These cell types were identified as follows: goblet cells (MUC2, ITLN1, HES6), gland mucous cells (GMCs; markers: OLFM4, SPINK4, MSMB), cancer cells (CEACAM6, CEACAM5, ALDH1A2), pit mucous cells (PMCs; GNK1, MUC5AC, TFF1), chief cells (PGC, PGA3, PGA5), proliferative cells (TOP2A, MKI67, BIRC5), macrophages (CSF1R, CD68, CD163), and fibroblasts (PDGFRB, DCN, COL1A1) (Figure 7C). Figure 7D illustrates the expression of the most specific cell markers for each respective cell type. Significant expression of CD163 by macrophages indicate their phenotype as alternative anti-inflammatory M2 macrophage. The TME-Met risk genes predominantly exhibited expression in macrophages and fibroblasts (Figure 7E). In particular, we observed the expression of the mentioned downregulated genes, such as GFRA1, GPX3, and SNCG, in fibroblasts, and PDE1B and CD36 in macrophages. Additionally, MAGEA3, CPVL, KYNU, and GPX3 showed expression in other cell types beyond macrophages and fibroblasts. Moreover, our analysis revealed cell-specific expressions, such as CARD11 in cancer cells, PNMA2 in proliferative cells, MAGEA3 in gland mucous cells, and KIT in chief cells. These findings highlight the diverse and context-specific expression patterns of the TME-Met risk genes across different cell types, shedding light on their potential roles and interactions within the tumor microenvironment.

To strengthen these observations, we further evaluated the expression pattern of these genes in another single-sequencing dataset (GSE112302) comprising 5 GC patients and 22, 464 cells. Following quality control and standardization, 10 clusters of cells were identified which were then renamed according to the standard single-cell markers as follows: T cells (CD3D), B cells (MS4A1), plasma cells (CD27), macrophage (CD68), epithelial cells (EPCAM), endothelial cells (CD34), fibroblasts (DCN), and mast cells (TPSAB1) (Figure 7C). Cell annotation in our analysis was consistent with the original study and the evaluation of the TISCH database, confirming a significant representation of the tumor microenvironment (TME) component (Jeong et al., 2021; Sun et al., 2021). Again, the TME-Met risk genes were predominantly expressed in macrophages and fibroblasts, particularly GPX3, CD36, KYNU, VCAN, and CPVL (Figure 7E).

**FIGURE 8:**
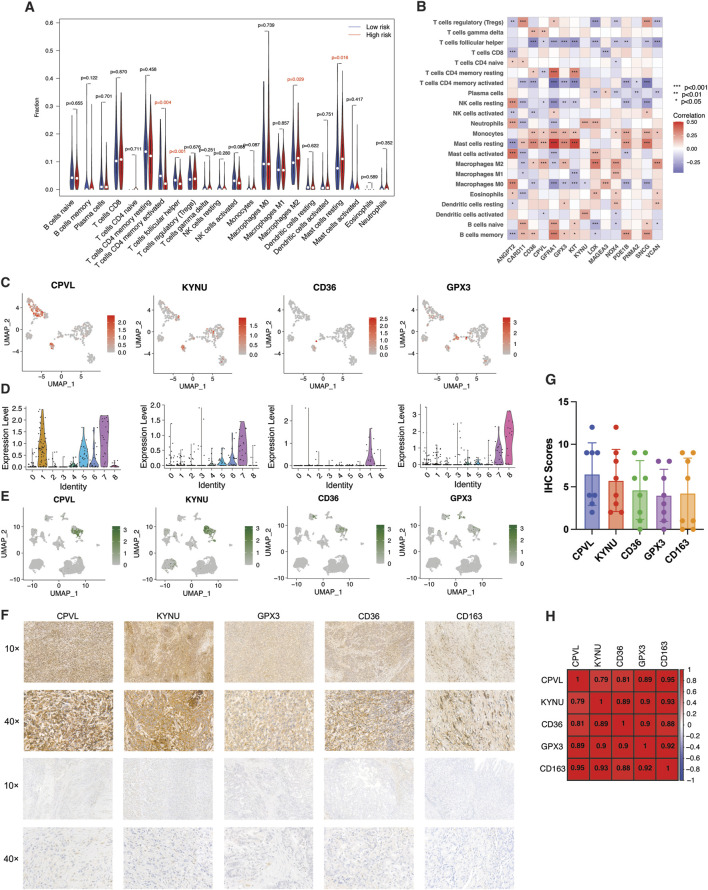
Identification and validation of M2 macrophage-related risk genes. **(A)** Violin plot of abundance of 22 subtypes of immune cells in risk subgroups. **(B)** Spearman’s correlation between infiltration of 22 subtypes of immune cells and individual TME-Met PI genes (n = 15) in TCGA STAD cohort. *p* values are shown as: *p < 0.05; **p < 0.01; ***p < 0.001. **(C)** UMAP plots and **(D)** Violin plots showing expression patterns of CPVL, KYNU, CD36, and GPX3 in single-cell gastric cancer dataset (GSE112302). **(E)** UMAP plots showing expression patterns of CPVL, KYNU, CD36, and GPX3 in GSE167297 dataset. **(F)** Representative images of expression (brown, cell cytoplasmic/nucleus stain) and **(** **G** **)** IHC quantification of expression level of CPVL, KYNU, CD36, and GPX3 and marker of M2 macrophage (CD163) in the clinical samples of stomach adenocarcinoma (n = 8). **(** **H** **)** Pearson’s correlation of expression level of CPVL, KYNU, CD36, and GPX3 and marker of M2 macrophage (CD163) in the clinical samples of stomach adenocarcinoma.

Next, we employed the CIBERSORT algorithm to investigate the correlation of fractions of 22 immune cell infiltrations in the gastric cancer microenvironment with TME-Met PI. The high-risk subgroup was highly infiltrated by M2 macrophage, and resting Mast cells (Figure 8A). Again, the aforementioned downregulated genes (CD36, CPVL, GFRA1, GPX3, KIT, PDE1B, and SCNG) were positively correlated with infiltration of monocytes, resting mast cells and M2 macrophages (Figure 8A). M2 macrophage observed significant positive correlation with CD36, CPVL, GPX3, LOX, NOX4, and VCAN (Figure 8B). Based on the results from CIBERSORT and single-cell analysis, 4 TME-Met prognostic genes, including CD36, CPVL, KYNU, and GPX3, showed strong association with M2 macrophages (Figures 8B–D). We selected these 4 genes for further immunohistochemical analysis to confirm this association (Figures 8E,F). There was a positive correlation identified between these 4 genes and the expression of M2 macrophage marker (CD163) in clinical specimens of gastric adenocarcinoma patients (n = 8). Although, an inverse relationship between CPVL and CD36 and KYNU indicating diverse functional phenotype of M2 macrophage in the TME (Figure 8G). Overall, these findings provide valuable insights into the complex interplay between TME-Met genes and immune cell infiltrations, specifically highlighting the involvement of M2 macrophages in the gastric cancer microenvironment.

### Therapeutic response prediction

**FIGURE 9:**
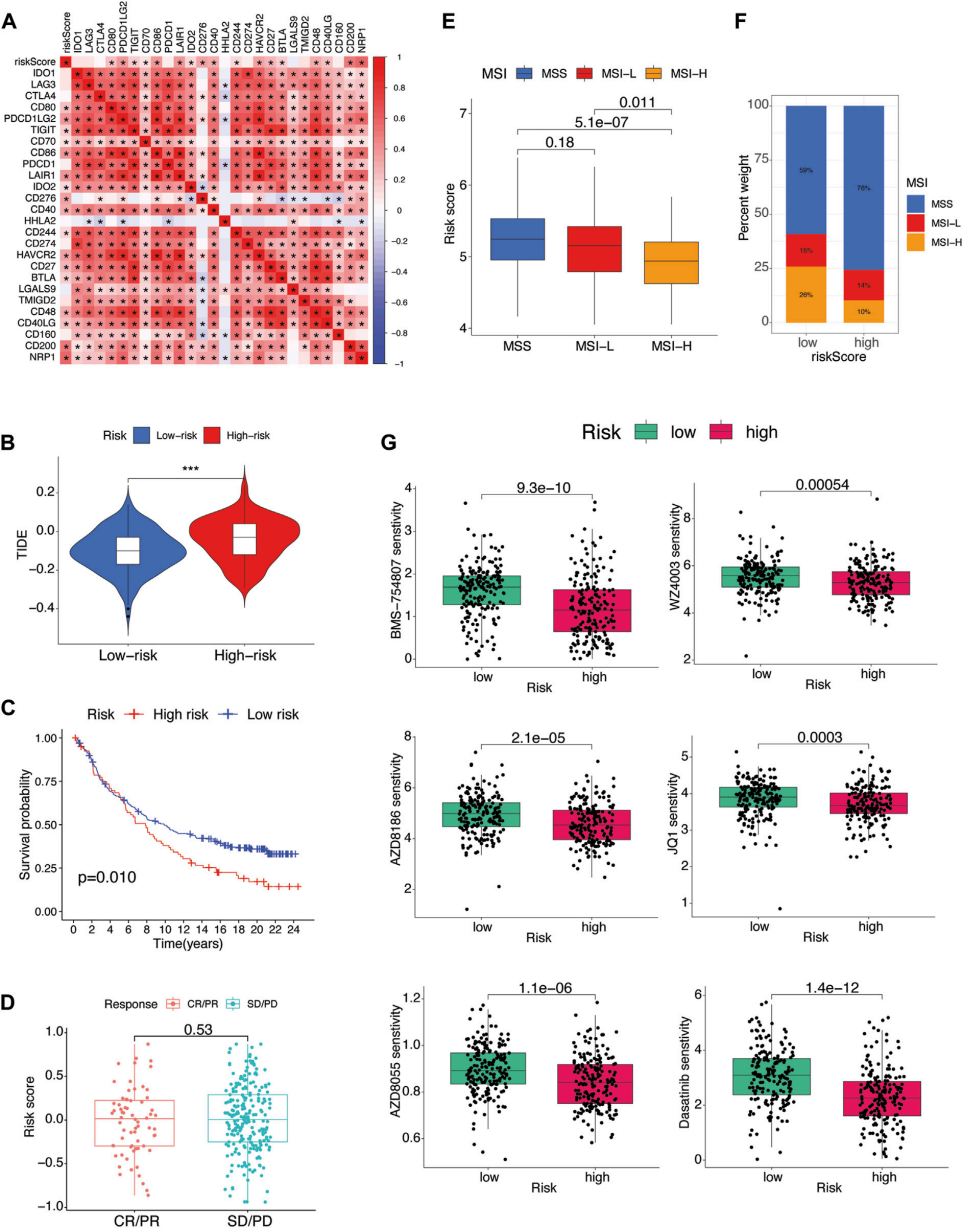
Therapeutic implications **(A)** Pearson’s correlation between riskScore and several immune checkpoint inhibitors. *p* values are shown as: *p < 0.05; **p < 0.01; ***p < 0.001. **(B)** Association between riskScore and TIDE score of TCGA STAD patients. **(C)** The Kaplan-Meier curves of difference in survival probability between risk subgroups and **(D)** boxplots of riskScore variation in responsiveness to immune checkpoint blockade of IMvigor210 urothelial carcinoma cohort. **(E)** Box plot of association of microsatellite instability (MSI) and riskScore. **(F)** Percent of MSI types in each risk subgroup. **(G)** Drug sensitivity analysis of risk subgroups.

**FIGURE 10:**
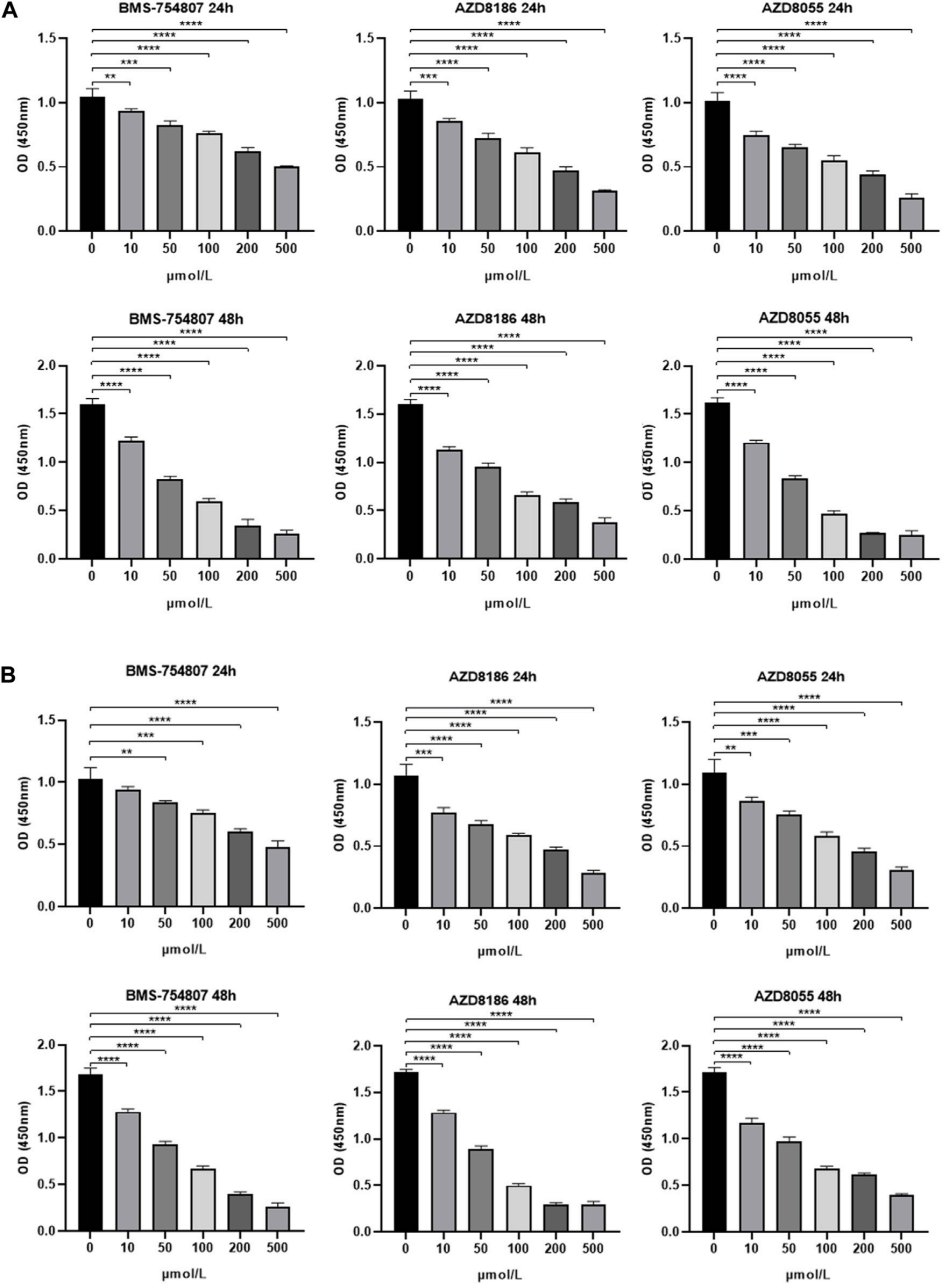
Experimental validation of drug sensitivity analysis **(A)** 24-h/48-h inhibitory capacity of BMS-754807, AZD8186, and AZD8055 on MKN45 and **(B)** MKN28 gastric cancer cells at various concentrations (0, 10, 50, 100, 200, 500 μmol/L) using CCK-8 assay. 0 μmol/L was considered as control. *p < 0.05; **p < 0.01; ***p < 0.001, ****p < 0.0001.

In the previous result sections, we have reported that there was no difference in infiltration of T cells including CD8 T cells deemed critical for immunotherapy due to expression of immune exhaustion markers such as PD-1, PD-L1 and CTLA4. As anticipated, the TME-Met riskScore failed to show any correlation with these established biomarkers of immune checkpoint inhibitors (Figure 9A). Simultaneously, lack of response to immunotherapy was also expected as reported in Figures 9B–D. The Tide score was high for high-risk subgroup indicating low likelihood of response to ICB (Figure 9B). Consequently, integration of urothelial carcinoma patients from IMvigor210 cohort treated with pembrolizumab showed significantly lower survival for high-risk subgroup indicating resistance to immunotherapy (Figure 9C). Although, no difference in response was observed (Figure 9D). Moreover, gastric cancer patients with high microsatellite instability (MSI-H) have reported low response to chemotherapy in localized resectable cases with no impact on prognosis (Pietrantonio et al., 2019; Vos et al., 2022). Our results indicated an inverse relationship between MSI and riskScore indicating the high-risk subgroup may respond to conventional chemotherapy; however, high-risk subgroup in our study comprised GC patients with advanced disease (Figures 9E,F). Conversely, the mesenchymal phenotype of gastric cancer has been associated with high resistance to chemotherapy (Cristescu et al., 2015; Oh et al., 2018). Therefore, we further explored the sensitivity of patients in the high-risk subgroup to 198 small molecules. Among these, six agents, including BMS-754807 (an inhibitor of insulin-like growth factor type I receptor [IGF-1R]), WZ4003 (NUAK1/2 inhibitor), AZD8186 (PI3K inhibitor), JQ1 (BET inhibitor), AZD8055 (an ATP-competitive mammalian target of rapamycin kinase inhibitor [mTOR]), and Dasatinib (a Src inhibitor), showed promise as potential treatment options. Out of these six identified drugs, four exhibited a high level of sensitivity, as indicated by their statistical significance. Given that Dasatinib has previously been evaluated in the context of gastric cancer, we selected three of these drugs for experimental validation. We employed the CCK-8 assay to assess the inhibitory effects of three highly sensitive drugs (BMS-754807, AZD8186, AZD8055) on gastric cancer cells, specifically the MKN45 and MKN28 cell lines. This was accomplished by subjecting MKN45 and MKN28 cancer cells to varying concentrations of these drugs for 1 and 2 days. The degree of drug inhibition was evaluated by measuring the optical density (OD) value, with a lower OD value indicating more effective inhibition compared to the control group treated with 0 μmol/L. As shown in Figure 10, all three drugs exhibited a dose-dependent effect on inhibiting MKN45 and MKN28 cells, and this effect became more pronounced after 48 h.

## Discussion

**FIGURE 11:**
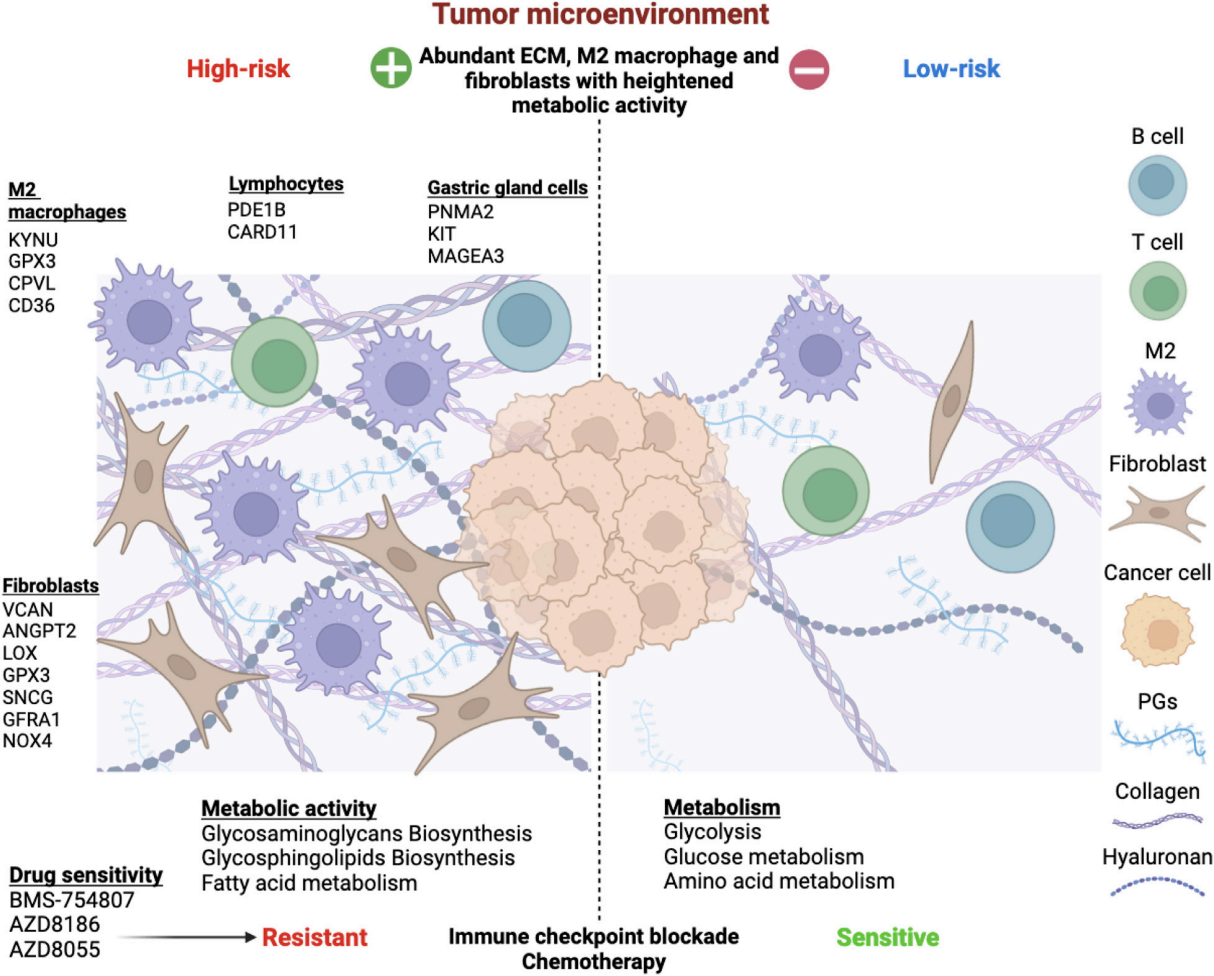
Overview of the risk stratification of gastric cancer patients based on tumor microenvironment and metabolism interplay. Extensive analysis revealed 15 risk genes expressed by diverse TME components. M2 macrophages upregulated KYNU, GPX3, CPVL, and CD36. Fibroblasts expressed VCAN, ANGPT2, LOX, GPX3, SNCG, GFRA1, and NOX4, impacting proliferation, mesenchymal transition, angiogenesis, and ROS production. PDE1B and CARD11 were associated with B and T lymphocytes, while PNMA2, KIT, and MAGEA3 were expressed by various gastric gland cells, such as chief cells, gland mucous cells, and proliferative cells. The TME-Met Interplay upregulated ECM biosynthesis and fatty acid metabolism. The high-risk subgroup showed resistance to immunotherapy and chemotherapy but responded to three molecular targeted drugs. Conversely, low-risk patients exhibited enriched glycolysis, glucose, and amino acid metabolism, with lower ECM content and sensitivity to immunotherapy and chemotherapy.

Mounting evidence has signified the importance of TME in the growth and development of cancer cells and resistance to cancer therapy. Each component of TME, namely, immune cells, stromal cells, blood vessels, extracellular matrix and signaling molecules, has been increasingly assessed for their pro-tumorigenic effects and potential for cancer therapy. In this study we evaluated the TME landscape of gastric cancer in the context of metabolic activity to estimate the probable crosstalk between the two dimensions. Based on the transcriptomic analysis of TME and metabolic-related genes in STAD, we were able to identify a cross-talk between these characteristics that was verified for protein-level interactions and prognosis. STAD samples were categorized into two molecular subtypes based on the expression patterns of these cross-talk genes. These two molecular subtypes had major differences in terms of prognosis, functional features, metabolic activity, and infiltration of immune cells (Figure 11). We further designed a prognostic index comprised of 15 genes which was derived from the upregulated genes between the subtypes using LASSO regression. The high-risk subgroup showed higher infiltration of fibroblast, M2 macrophages, and resistance to cancer therapy.

Gastric cancers (GCs) can be categorized histologically using the Lauren and WHO systems (Lauren, 1965; Hu et al., 2012). Lauren classifies them as either intestinal or diffuse, while WHO categorizes them into papillary, tubular, mucinous, and poorly cohesive subtypes. However, these classifications, though influential in treatment decisions, do not adequately address the heterogeneity of GCs for improved personalized patient care (Dicken et al., 2005). The development of genomic sequencing technology has led to the emergence of molecular subtyping, with a seminal study conducted by The Cancer Genome Atlas (TCGA) research team identifying four molecular subtypes of GC: EBV-positive, microsatellite-unstable, genomically stable, and chromosomal instability (CIN) (The Cancer Genome Atlas Research Network, 2014). Other studies have also reported molecular subtypes of GC with similar characteristics, including one closely resembling our high-risk subgroup, the mesenchymal type (Lei et al., 2013; Cristescu et al., 2015; Oh et al., 2018). In these studies, the mesenchymal subtype was characterized by a diffuse histological variant, genomic stability, low mutation rates, a high recurrence rate, poor prognosis, and resistance to chemotherapy (Lei et al., 2013; Cristescu et al., 2015; Oh et al., 2018). Our single-cell analysis supports the attribution of high risk subgroup as a mesenchymal phenotype due to absence of risk gene expression in primarily epithelial-origin cancer cells. Additionally, our findings could also be interpreted as a blend of mesenchymal and metabolic subtypes as identified by Lei et al. (Lei et al., 2013). These subtypes exhibited enrichment in KEGG pathways (mesenchymal: focal adhesion, ECM-receptor interaction; metabolic: various metabolic pathways) and GO terms (mesenchymal: cell adhesion; metabolic: digestion), all of which were collectively expressed in our pathway enrichment analysis (Lei et al., 2013). Indeed, their study demonstrated heightened *in vitro* sensitivity of the mesenchymal subtype to phosphatidylinositol 3-kinase-AKT-mTOR inhibitors, aligning with our findings. Furthermore, mesenchymal phenotype (MP) subtypes exhibited greater responsiveness to IGF1/IGF1R pathway inhibition compared to epithelial subtypes, consistent with our results (Oh et al., 2018). Additionally, GC subtyping based solely on TME features and metabolic reprogramming has identified subtypes resembling our high-risk subgroup, particularly characterized by heightened GAGs metabolism and M2 macrophage infiltration (Chen et al., 2023; Cho et al., 2018; Zhu et al., 2021; Chen et al., 2022; Han et al., 2022; Tao et al., 2023). However, these approaches heavily rely on the tumor component of gastric cancer for genetic mutations and metabolic activity related to glycolysis compromising the unraveling of TME critical role in these GC subtypes. In contrast, our study introduces a novel approach, excluding tumor content and focusing solely on the tumor microenvironment components to explore other cancer hallmarks, such as metabolism, providing new insights into GC biology and potential therapeutic strategies.

There were two cluster of patients identified when the TME and metabolic cross-talk DEGs with prognostic significance were subjected to consensus clustering. The two clusters had major differences from the perspective of tumorigenesis, metabolism and immunology. The cluster with worst prognosis exhibited a stronger TME features mainly in terms of stromal components. Metabolically, metabolism of major glycosaminoglycans (GAGs), namely, chondroitin sulfate (CS), keratan sulfate (KS), and heparan sulfate (HS), were upregulated which are ubiquitous structural and functional components of extracellular matrix (ECM) and has been associated with cancer malignancy (Cui et al., 2013). Biosynthesis of CS was identified to be active in several cancer types compared with normal tissues (Shi et al., 2019). CS was shown to inhibit PTEN, a tumor suppressor gene, leading to the activation of melanoma cell proliferation (Lin et al., 2018). An abnormal increase in KS levels was strongly correlated with enhanced proliferation in various tumors, including lymphoma, astrocytic tumors, and glioblastoma (Kato et al., 2008; Nakayama et al., 2013; Hayatsu et al., 2008). Sulfated KS was shown to trigger the activation of the MAPK and PI3K pathways in lymphoma cells, thereby initiating growth signals (Nakayama et al., 2013). In fact, our results also indicated the activation of PI3K pathway as determined by the DEGs between the clusters suggesting that upregulated GAGs metabolism may exert their downstream effects via PI3K activation. Likewise, HS, expressed on cell surfaces and within the extracellular matrix, was found to serve as a receptor for a growth-related ligand, thereby promoting cancer growth (Bat-Erdene et al., 2018). GAG abundance was also shown to promote metastasis of renal cell carcinoma (Gatto et al., 2016). Elevated HS biosynthesis was correlated with cancer cell migration by interacting with growth factors and regulation of the epithelial-to-mesenchymal transition (EMT) (Itano et al., 2002). Biosynthesis of glycosphingolipids (GSLs) of ganglio-series and globo-series were also upregulated which are mainly involved in cell-cell interactions and signal transduction pathways (Hakomori, 2003). GSLs are also implicated in oncogenic transformation and have been served as cancer biomarkers (Guan et al., 2009). In fact, therapeutic potential of GSLs in gastric adenocarcinoma was shown by targeting the glycosphingolipid globotriaosylceramide (Gb3/CD77) with shiga toxin B-subunit (STxB), which was expressed by 72% of cases (Geyer et al., 2016). Overall identification of these targets provides an opportunity for therapeutic investigations aimed at the TME features which rather show superior prognostic potential as compared to tumor cells.

On the other hand, the patients in the cluster 1 had major tumor component and were enriched in protein and glucose metabolism. The lower enrichment of these pathways in Cluster 2 merely indicates the low tumor content and lower cellular content of TME. Relevant cellular content of the TME of cluster 2 in the context of our study was identified as mast cells, macrophages and fibroblasts. The risk genes that belonged to TME and metabolism were expressed by macrophages and fibroblasts as confirmed by CIBERSORT results, single cell analysis and IHC evaluation. M2 macrophage has previously been identified as the most prominent immune cell associated with immunosuppressive TME, progression, and prognosis of gastric cancer (Yamaguchi et al., 2016; Piao et al., 2022). Of the risk genes, 4 genes were evidently expressed by M2 macrophages, namely, GPX3, CPVL, CD36, and KYNU. Extracellular glutathione peroxidase (GPX3) was downregulated in cancer samples but was upregulated in the cluster 2 patients with higher TME content and metabolic activity, and is considered a prognostic gene in gastric patients (Chang et al., 2020). However, it has shown a dual role in various cancers including gastric cancer (Chang et al., 2020). Knockdown of GPX3 in gastric cancer was shown to result in tumor cell invasion and migration (Cai et al., 2019). Our study indicates that its prognostic significance may arise from its expression in the TME specifically M2 macrophages and fibroblasts. Kynureninase (KYNU), a hydrolase involved in tryptophan metabolism, was also expressed by these two cells. KYNU has been identified as a novel transcriptional target of CD44-downstream signaling and underpins CD44-promoted breast tumor cell invasion (Al-Mansoob et al., 2021). CD44 interaction with Hyaluronic acid (HA), one of the major GAGs that is extensively studied in cancer, has been associated with tumor cell proliferation and enhancing chemo resistance via regulating PI3K/Akt and MAPK signal pathways (Toole and Slomiany, 2008; Chanmee et al., 2015). Although, HA was not upregulated in cluster 2, other GAGs were significantly upregulated implicating their possible role in upregulation of KYNU in gastric cancer. CPVL (Carboxypeptidase Vitellogenic Like), a novel serine carboxypeptidase, was originally characterized in macrophages and its functions may include digestion of phagocytosed particle within lysosomes, contribution to an inflammatory protease cascade, and participation in peptide trimming for antigen presentation (Mahoney et al., 2001). In cancer, it has been recognized as an oncogene contributing to cancer progression and therapeutic resistance (Zhu et al., 2023; Yang et al., 2021). It inhibited the glioma cell apoptosis by interacting with BTK and downregulating STAT1 phosphorylation through the facilitation of p300-mediated STAT1 acetylation (Yang et al., 2021). While it facilitated resistance to CDK4/6 inhibitors in breast cancer (Zhu et al., 2023). Our results indicate the CPVL expressed by macrophages/fibroblasts may also contribute to cancer progression which needs further investigations. CD36 is a scavenger receptor that performs various important functions in cancer such as regulating lipid uptake, immune recognition, inflammation, adhesion, and cell death in various cells (Wang and Li, 2019). CD36 has been reported to promote GC progression, metastasis and prognosis mainly involving its role in lipid uptake and promotion of fatty acid oxidation (Pan et al., 2019; Jiang et al., 2019b). In the context of macrophages, it was shown that CD36-mediated metabolic crosstalk between tumor cells and macrophages could promote liver metastasis (Yang et al., 2022). These results imply that CD36 expression by macrophages in gastric cancer may also involve such metabolic crosstalk in promoting the GC metastasis, specially involving fatty acids which was also upregulated in high-risk subgroup. Overall, these 4 genes are intricately linked to metabolic activity of the ECM involving macrophages and fibroblasts in promoting the gastric cancer progression and metastasis. Further evaluation of these genes in the aforementioned context may unravel their potential for targeted therapy.

In addition to the four genes strongly associated with M2 macrophages, GPX3, VCAN, ANGPT2, LOX, SNCG, GFRA1 and NOX4 were notably expressed in fibroblasts. VCAN, a chondroitin sulfate proteoglycan, plays a pivotal role in tumorigenesis, with increased expression linked to various cancers and poor prognosis (Mitsui et al., 2017). Consistent with our findings, upregulation of VCAN was observed in stromal and epithelial compartments of high-grade serous ovarian tumors and TGF-β-treated normal ovarian fibroblasts (Yeung et al., 2013). Co-culture experiments further demonstrated that VCAN upregulation in CAFs enhanced the aggressiveness of ovarian cancer cells. ANGPT2, acting as a context-dependent antagonist, can disrupt angiopoietin-1-induced Tie2 phosphorylation, promoting angiogenesis (Fukumura et al., 2018). Its high expression in various tumor cells underscores its pivotal role in tumor angiogenesis and inflammation, rendering it an appealing target for vascular therapy (Scholz et al., 2015). Its upregulation in fibroblasts indicate the role of fibroblasts in promoting angiogenesis in gastric cancer. LOX, a secreted copper-dependent amine oxidase, expressed by various cells including fibroblasts, primarily functions in crosslinking collagens and elastin (Wang et al., 2016). Its overexpression in cancer has been associated with malignant progression, with reports indicating its promotion of epithelial-mesenchymal transition (EMT) in gastric cancer under hypoxic conditions (Kasashima et al., 2016). SNCG, primarily expressed in neural tissues, is reported to upregulate in cancer tissues such as breast, ovary, colon, liver, and cervical cancer (Liu et al., 2005). Like LOX, it has been primarily reported for regulating EMT to promote cancer metastasis (Hsu et al., 2020; Liu et al., 2022). GFRA1, interacting with glial cell-derived neurotrophic factor (GDNF), promotes tumor progression (Cavel et al., 2012; Ni et al., 2022). Tumor-associated macrophage-derived GDNF facilitates gastric cancer liver metastasis via GFRA1-mediated autophagy flux (Ni et al., 2023). Similarly, perineural invasion of pancreatic cancer involves endoneurial macrophages secreting GDNF and activating RET tyrosine kinase receptor, a GFRA1 coreceptor (Cavel et al., 2012). These findings suggest potential macrophage-fibroblast crosstalk in gastric cancer through GFRA1-GDNF signaling. The notable metabolic gene in fibroblasts was the (NOX4) NADPH Oxidase 4, which contributes to elevated reactive oxygen species (ROS) levels. NOX-derived ROS production is linked to diverse cancerogenic processes through induction of redox-sensitive transcription factors such as HIF-1α, NFκB and NRF2 (Szanto, 2022). In summary, fibroblasts play a critical role in gastric cancer progression by promoting proliferation, metastasis, and angiogenesis through the upregulation of these markers. Consequently, these markers represent important therapeutic targets warranting further investigation, particularly with refined patient selection criteria.

Phosphodiesterase 1 (PDE1s: PDE1A; PDE1B; PDE1C) targets second messengers (cAMP and cGMP) to regulate diverse physiological processes, with limited exploration in cancer. PDE1B is overexpressed in lymphoblastoid B-cells and leukemic cell lines of B- (RPMI-1788, Daudi) and T-(MOLT-4, NA, Jurkat) cell origin. Upregulation in human peripheral blood lymphocytes (HPBL) upon mitogenic stimulation suggests a role in proliferation. Inhibition has demonstrated reduced cell growth and induced apoptosis in leukemic cells (Jiang et al., 1996). CARD11 is another gene that is mainly expressed in lymphoid tissues and associated with B and T cell lymphomas (Kataoka et al., 2015). Our study also indicated its positive correlation with naïve and memory B cells and regulatory T cells, and single-cell analysis confirmed its enrichment in B and T cells. PNMA2, primarily expressed in the brain, is associated with paraneoplastic neurological syndromes, often accompanying peripheral solid tumors. Antibodies to PNMA proteins can serve as diagnostic markers for specific cancers (Xu et al., 2024). KIT regulates various cellular processes including growth, survival, migration, differentiation, and secretion. Amplification and activating mutations in KIT are frequently observed in gastrointestinal stromal tumors (GISTs) and melanoma (Miettinen and Lasota, 2005). MAGEA3, which was expressed in all cells including tumor cells, is a Cancer Testis Antigen (CTA) and reported in almost all types of cancer and is considered a promising candidate for immunotherapy (Das et al., 2021). PNMA2, KIT, and MAGEA3 are predominantly expressed by proliferative cells (PCs), Chief cells and gland mucus cells (GSCs), respectively.

The TME-Met prognostic gene signature was demonstrated to resist to immunotherapy and hence must be explored for alternative therapeutic targets. The various aspects of our outcomes present us with such opportunities. For example, GAGs and GSLs have been explored as cancer biomarkers and therapeutic targets (Yip et al., 2006; Edwards, 2012; Furukawa et al., 2019; Wei et al., 2020; Yu et al., 2020). Likewise, each risk gene could be exploited for therapeutic investigations, particularly in the context of M2 macrophage such as CD36 (Yang et al., 2022). We have identified six potential molecular targeted agents with therapeutic potential for this patient subset. Among these agents, inhibitors targeting IGF-1R (BMS-754807) and the PI3K-mTOR pathways (AZD8186, AZD8055) emerged as the most promising candidates for therapeutic intervention in gastric cancer. Notably, BMS-754807, a selective IGF-1R inhibitor, exhibited potent inhibitory effects on gastric cancer cells. This aligns with a previous study demonstrating the activation of the IGF1/IGF1R pathway in mesenchymal gastric tumors, which displayed sensitivity to Linsitinib (OSI-906), another selective IGF-1R inhibitor (Oh et al., 2018). AZD8186, a selective PI3Kβ/δ inhibitor, has shown anti-tumor activity in PTEN-deficient preclinical models, and has undergone clinical testing among patients with advanced solid cancers including gastric cancer (Choudhury et al., 2022; Suh et al., 2023). Despite the good tolerability of the AZD8186 and paclitaxel combination, there was limited clinical effectiveness noted in advanced gastric cancer cases exhibiting PTEN loss. These results stress for the enhanced patient selection such as the high-risk subgroup in our study may benefit from AZD8186 due to enrichment in PI3K pathway as compared to low-risk subgroup. Likewise, mTOR inhibitors such as 2,6-DMBQ (AZD8055) has also been previously reported for their inhibitory efficacy in gastric cancer (Zu et al., 2020). Mechanistically, AZD8055 inhibits mTORC1 substrates p70S6K and 4E-BP1, and mTORC2 substrate AKT, suppressing downstream proteins. *In vitro*, it hampers proliferation and induces autophagy in H838 and A549 cells. *In vivo*, AZD8055 hinders tumor growth by modulating phosphorylated S6 and AKT levels in a dose-dependent manner. Our results can further provide useful input in gastric cancer patient selection for this proposed treatment. These findings indicate that inhibiting IGF-1R and the PI3K-mTOR pathway can significantly influence gastric cancer prognosis by modulating metabolic and TME features. Additional agents, such as the BET inhibitor (JQI) and src inhibitor (Dasatinib), have also demonstrated promising preclinical efficacy in gastric cancer (Zhou et al., 2020; Wang et al., 2022). JQI has been effective in suppressing metastasis, while Dasatinib has shown potential in sensitizing cancer cells to chemotherapy (Zhou et al., 2020; Wang et al., 2022). Notably, these two features were found to be predominant in the high-risk subgroup of patients. Consequently, by carefully selecting patients from this subgroup, the effectiveness of these agents could potentially be further enhanced in the treatment of gastric cancer.

Our study has several limitations. The accuracy and generalizability of the findings depend heavily on the quality and representativeness of the data. Datasets were used from only three distinct populations which may have limitations in reflecting the diversity of gastric cancer patients. While transcriptomic analysis is a powerful tool, it has its limitations. It provides information on gene expression levels but does not account for post-translational modifications or protein activity, which can be crucial in understanding the functional role of genes in cancer progression. To partially address this limitation, we made efforts to validate the interactions of these genes at the protein level by consulting external databases and subsequently confirming these findings in a limited cohort of gastric cancer clinical samples. However, comprehensive validation through prospective clinical studies is imperative to ascertain the clinical significance of the identified molecular subtypes and prognostic genes. Our study fails to provide deep insights into the mechanistic details of how these risk genes drive the progression of gastric cancer. Hence, in-depth functional studies should be carried out to elucidate the exact mechanisms by which the identified genes and pathways contribute to gastric cancer progression and treatment resistance. The study suggests several potential therapeutic targets, such as GAGs, GSLs, and specific genes like CD36, CPVL, GPX3 and KYNU. These targets should be further explored for the development of targeted therapies, including small molecules, antibodies, or other treatment modalities.

## Conclusion

By investigating tumor microenvironment (TME) characteristics, especially in the context of metabolic reprogramming, we have uncovered promising opportunities for enhancing gastric cancer diagnosis and treatment strategies. The identification of molecular subtypes and development of a 15-gene prognostic signature, encompassing significant molecular and functional differences, offers a valuable tool for predicting patient outcomes and guiding personalized treatment approaches. However, further research and clinical trials are necessary to validate and optimize these findings for clinical applications.

## Data Availability

The original contributions presented in the study are included in the article/Supplementary Material, further inquiries can be directed to the corresponding authors.
